# Individualised short-term therapy for adolescents impaired by attention-deficit/hyperactivity disorder despite previous routine care treatment (ESCAadol)—Study protocol of a randomised controlled trial within the consortium ESCAlife

**DOI:** 10.1186/s13063-018-2635-2

**Published:** 2018-04-27

**Authors:** Julia Geissler, Thomas Jans, Tobias Banaschewski, Katja Becker, Tobias Renner, Daniel Brandeis, Manfred Döpfner, Christina Dose, Christopher Hautmann, Martin Holtmann, Carolin Jenkner, Sabina Millenet, Marcel Romanos

**Affiliations:** 1 Department of Child and Adolescent Psychiatry, Psychosomatics and Psychotherapy, University Hospital of Würzburg, Center of Mental Health, Margarete-Höppel-Platz 1, 97080 Würzburg, Germany; 2 Department of Child and Adolescent Psychiatry and Psychotherapy, Central Institute of Mental Health, Medical Faculty Mannheim, University of Heidelberg, Mannheim, Germany; 3 Department of Child and Adolescent Psychiatry, Psychosomatics and Psychotherapy, Medical Faculty of the Philipps-University Marburg and the University Hospital Marburg, Marburg, Germany; 4 Department of Child and Adolescent Psychiatry and Psychotherapy, University Hospital Tübingen, Tübingen, Germany; 5 Department of Child and Adolescent Psychiatry and Psychotherapy, Psychiatric Hospital, University of Zürich, Zurich, Switzerland; 6 Zurich Center for Integrative Human Physiology, University of Zürich, Zurich, Switzerland; 7 Neuroscience Center Zurich, University and ETH Zürich, Zurich, Switzerland; 8 Department of Child and Adolescent Psychiatry, Psychosomatics and Psychotherapy, Medical Faculty of the University of Cologne, Cologne, Germany; 9 School of Child and Adolescent Cognitive Behaviour Therapy (AKiP), University Hospital of Cologne, Cologne, Germany; 10 LWL-University Hospital Hamm, Ruhr-University Bochum, Hamm, Germany; 11 Clinical Trails Unit at University Medical Center Freiburg, Freiburg, Germany

**Keywords:** Attention-deficit/hyperactivity disorder, ADHD, Adolescents, Behaviour therapy, Individualised modular treatment programme, Telephone-assisted self-help, RCT

## Abstract

**Background:**

Despite the high persistence rate of attention-deficit/hyperactivity disorder (ADHD) throughout the lifespan, there is a considerable gap in knowledge regarding effective treatment strategies for adolescents with ADHD. This group in particular often shows substantial psychosocial impairment, low compliance and insufficient response to psychopharmacological interventions. Effective and feasible treatments should further consider the developmental shift in ADHD symptoms, comorbidity and psychosocial adversity as well as family dysfunction. Thus, individualised interventions for adolescent ADHD should comprise a multimodal treatment strategy. The randomised controlled ESCAadol study addresses the needs of this patient group and compares the outcome of short-term cognitive behavioural therapy with parent-based telephone-assisted self-help.

**Methods/design:**

In step 1, 160 adolescents aged 12 to 17 years with a diagnosis of ADHD will undergo a treatment as usual (TAU) observation phase of 1 month. In step 2, those still severely affected are randomised to the intervention group with an Individualised Modular Treatment Programme (IMTP) or a telephone-assisted self-help programme for parents (TASH) as an active control condition. The IMTP was specifically designed for the needs of adolescent ADHD. It comprises 10 sessions of individual cognitive behavioural therapy with the adolescents and/or the parents, for which participants choose three out of 10 available focus modules (e.g. organisational skills and planning, emotion regulation, problem solving and stress management, dysfunctional family communication). TASH combines a bibliotherapeutic component with 10 counselling sessions for the parents via telephone. Primary outcome is the change in ADHD symptoms in a clinician-rated diagnostic interview. Outcomes are assessed at inclusion into the study, after the TAU phase, after the intervention phase and after a further 12-week follow-up period. The primary statistical analysis will be by intention-to-treat, using linear regression models. Additionally, we will analyse psychometric and biological predictors and moderators of treatment response.

**Discussion:**

ESCAadol compares two short-term non-pharmacological interventions as cost-efficient and feasible treatment options for adolescent ADHD, addressing the specific needs and obstacles to treatment success in this group. We aim to contribute to personalised medicine for adolescent ADHD intended to be implemented in routine clinical care.

**Trial registration:**

German Clinical Trials Register (DRKS), Current Controlled Trial DRKS00008974, http://apps.who.int/trialsearch/Trial2.aspx?TrialID=DRKS00008974; http://www.drks.de/drks_web/navigate.do?navigationId=trial.HTML&TRIAL_ID=DRKS00008974; Registered on 28 December 2015.

**Electronic supplementary material:**

The online version of this article (10.1186/s13063-018-2635-2) contains supplementary material, which is available to authorized users.

## Background

Childhood attention-deficit/hyperactivity disorder (ADHD) shows a high tendency to persist into adulthood [1–3], causes significant social, emotional and academic impairment for the individual and poses a risk of failing to achieve important educational and psychosocial developmental goals [4]. Highly comorbid with subsequent adolescent and adult depression, anxiety and substance abuse, it is also a predisposing factor for delinquency [5, 6]. ADHD confers a substantial economic burden with high direct and indirect costs for society [7, 8]. There is a great need for effective, feasible and safe treatment strategies. However, the evaluation of treatment response in efficacy studies is typically based on highly selected patient groups, excluding patients with poor compliance, most comorbid conditions or common psychosocial adversity [9].

Psychopharmacological treatment options, especially psychostimulants and atomoxetine, have been studied extensively and their efficacy in targeting core symptoms of ADHD in children and adolescents has been validated by a large body of research [10–12]. In combination with psychoeducation, pharmacotherapy is recommended as treatment of choice for severe and impairing ADHD symptoms according to national and international guidelines [13]. However, the benefits of pharmacotherapy for adolescents might be reduced in patients with comorbid disorders [12, 14]. Since medication adherence especially in adolescence is poor [15, 16], non-pharmacological treatment options are indispensable.

While there is empirical support for the efficacy of psychosocial interventions in children [17] and adults [18], cognitive behavioural therapy (CBT) approaches in adolescent ADHD are comparatively scarce. A study assessing CBT in a group setting found a reduction in ADHD symptoms and functional impairment in the treatment group [19], whereas the usefulness of rational emotive therapy [20] in adolescents with ADHD could not be confirmed.

Especially controlled studies on individual psychotherapy in adolescents are largely lacking [21, 22]. To date, the available empirical evidence is limited to a small number of mostly uncontrolled studies and existing randomised controlled trials (RCTs) comprised exclusively og multicomponent interventions [12]. An unpublished Canadian trial [Mongia M, Hechtman L: Cognitive behaviour therapy in adolescents with attention deficit hyperactivity disorder: a pilot study, unpublished] reported a 14-session CBT intervention and additional coaching calls as an add-on to psychopharmacological treatment to result in a reduction of ADHD symptoms as well as improvements in self-esteem and level of disability. However, the small sample size (*N* = 18) and the lack of a control group are serious caveats of this study. Another study found an adaptation of CBT for adult ADHD to adolescents with residual symptoms under medication to bring about positive changes on a range of variables such as inattention, medication adherence, self-esteem, family and academic functioning (uncontrolled study [23]). There is some evidence for the efficacy of a mindfulness training for adolescents and their parents (uncontrolled study; [24]). The authors reported positive effects on attention and behavioural problems, executive functioning and parenting while stressing the need for maintenance strategies to sustain those effects.

Furthermore, there are demonstrated benefits of an organisational skills training and reinforcement of goal achievement for older children [25] and for adolescents in terms of ADHD symptoms and impairment in the framework of extensive summer treatment programmes or school-based interventions [21, 26], and a reduction of parent-teen conflicts through family-based interventions for adolescents and their parents [27]. The Supporting Teens’ Academic Needs Daily (STAND) programme demonstrated the superiority of a combination of family sessions and group sessions for parents to treatment as usual, targeting family cooperation by teaching both adolescents and parents with a focus on improving academic performance. The authors found improvements in academic functioning and ADHD symptoms [28]. Most recently, Boyer and colleagues [29–31] showed comparable positive effects of two non-ADHD-specific CBT interventions, Plan My Life versus Solution-Focused Treatment, in a randomised trial. Each intervention arm combined parent and adolescent sessions with motivational enhancement therapy.

The scarcity of (psycho-)therapy research for adolescent ADHD may partly be due to the low compliance seen in youths with ADHD [32]. Furthermore, the development and evaluation of general treatment strategies is complicated by the need for individualised treatment options taking into account the broad comorbid development, the shift in ADHD core symptoms, and psychosocial adversity as well as family dysfunction, e.g. poor parenting skills and parental mental health problems [7, 33, 34]. Previous trials conducted by our study group on treatment options for childhood and adult ADHD [35–38] and the development of interventions for adolescents with achievement problems including ADHD symptoms [39] underscored the feasibility of successfully conducting clinical trials with adolescent patients.

In sum, especially individualised treatment plans for adolescent ADHD need to be developed and evaluated combining psychosocial and pharmacological interventions in the framework of a multimodal treatment strategy. Treatment must take into account the prevalent compliance problems of youths with ADHD and must be individualised with respect to each patient’s constellation of impairment in terms of ADHD symptom expression and developmental comorbidity.

To address the needs of this group of patients, the ESCAadol study (Evidence-based stepped care of ADHD: individualised short-term therapy for adolescents impaired by ADHD despite previous routine care treatment) was devised. As adolescent patients with ADHD often present with comorbid diagnoses and severe impairment, the target group of ESCAadol are adolescents aged 12–17 years who are still severely impaired by their ADHD symptoms according to the *Diagnostic and Statistical Manual of Mental Disorders, Fifth Edition* (DSM-5) [40] despite having been in routine clinical care for at least 6 months.

Based on established interventions, we composed a short-term, Individualised Modular Treatment Programme (IMTP), adapting elements of dialectical behaviour therapy (DBT) for adolescents in general [41] and specifically for ADHD in adults [42] as well as interventions from the German treatment manuals for externalising disorders (THOP, [43]), performance problems in adolescents (SELBST, [44]), family interventions (PLAN-E, [45]) and social skills training (FESKO, [46]). We chose interventions targeting ADHD symptoms and related issues, such as emotion regulation and self-confidence, in addition to organisational difficulties, since these are emerging as critical issues in adolescence. For an outline of the focus areas included in the programme and their respective therapeutic targets, please refer to Table 3.

The IMTP consists of 10 sessions and addresses deficits in cognitive control, emotion regulation, organisation and planning, conflict management, medication compliance and parental mental health. The choice of a short-term treatment accounts for the poor therapy adherence and motivation often seen in adolescent ADHD, is supposed to be cost-effective, supports trial feasibility and facilitates broad dissemination. Different treatment modules will allow for the adaptation to specific impairment patterns. This programme will be evaluated in a multi-centre, randomised effectiveness study with an active control condition and blinded observer ratings of the primary endpoint (change in ADHD symptoms). Participants in the active control condition will be treated with a telephone-assisted self-help (TASH) programme for parents of adolescents with ADHD. Although child- or adolescent-centred interventions become more important the older the patient is, parenting interventions are still recommended for the treatment of ADHD in adolescents [47]. Self-help interventions have already been shown to have effects on parent-rated externalising behaviour problems in preschool- and school-aged children [48, 49]. Moreover, a pilot-study using a pre-post design found a large improvement in parent-rated ADHD symptoms during the TASH intervention used in this study [50]. We chose TASH as the active control condition since it was designed as a first step in routine care for unselected patient groups and we thus assume low to zero effectiveness in our highly affected and persistent clinical sample that has already received counselling and treatment. The therapeutic strategies applied offer sufficient additional support for the family while the patient remains in routine clinical care during study participation. Weekly assessments ensure patients’ safety.

Should this newly developed, highly individualised, short-term, non-pharmacological intervention prove to be effective in ameliorating adolescent ADHD symptoms, it will offer a cost-efficient economic and feasible treatment option which is designed to create high treatment motivation for a patient group with frequent compliance issues and may easily and broadly be implemented in clinical care.

Our study aims to add to the development of treatment strategies for adolescents and to the evidence base of treatment approaches. The study will furthermore investigate predictors and moderators of treatment response. This report presents Version 6 (20 December 2016) of the study protocol. We followed the Standard Protocol Items Recommendations for Interventional Trials (SPIRIT) Statement 2013 (see Additional file 1: SPIRIT 2013 Checklist: recommended items to address in a clinical trial protocol and related documents). For the complete World Health Organization Trial Registration Data Set, please see Additional file 2.

Our primary hypothesis is a significantly greater reduction in the primary outcome (ADHD symptoms) in the IMTP group compared to the active control condition TASH. On secondary outcomes such as global impairment, internalising and externalising symptoms, we expect greater benefit in the IMTP group compared to TASH, whereas parenting is expected to improve more in the parent-focused TASH group. We expect treatment effects and group differences to remain stable at follow-up.

## Methods/design

### Study design and trial flow

**Fig. 1:**
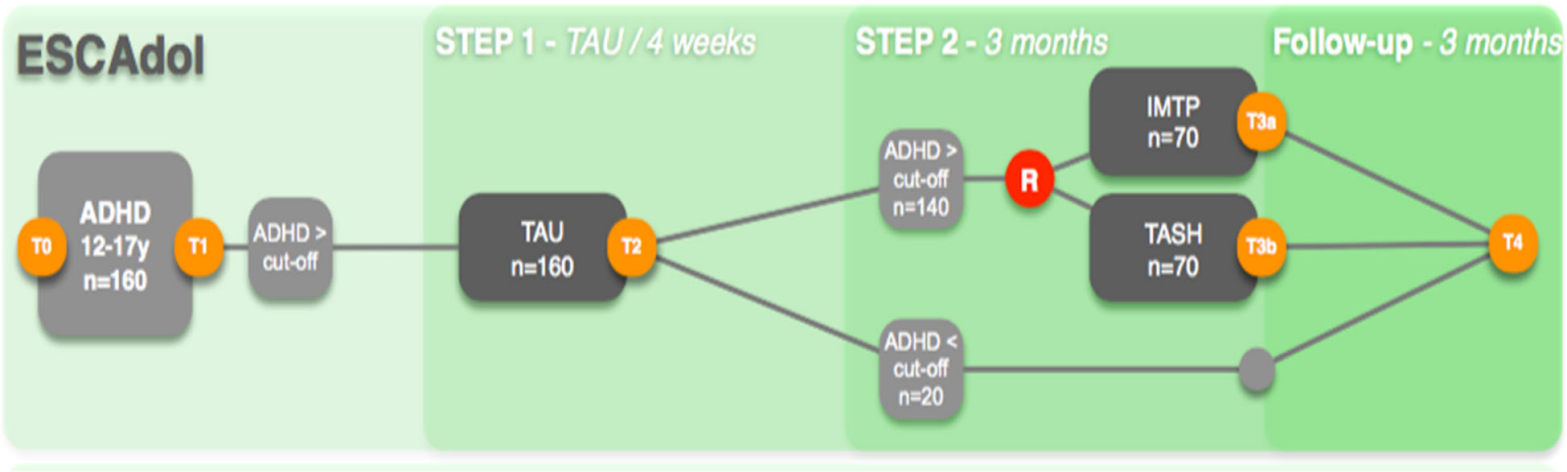
ESCAadol trial flow. *IMTP* = Individualised Modular Treatment Programme, *TASH* = telephone-assisted self-help, *TAU* = Treatment as usual, *R* = randomisation, *T0* = assessment for eligibility, *T1–T4* = study visits (outcome parameters)

Written informed consent for the main trial and all add-on investigations will be obtained by a senior member of the study team from all participating adolescents, parents and – if applicable – teachers. The study is designed as a randomised controlled, multi-centre trial. In *step 1* of the study, a planned number of *N* = 160 participating adolescents with ADHD will undergo a 4-week treatment-as-usual (TAU) phase with their attending physician in order to assess changes in ADHD symptoms in routine care. This is a strictly observational phase, all treatment decisions remain with the attending physician. At the end of the TAU phase, the study team will obtain information on which treatments have been implemented during TAU, followed by an assessment of ADHD severity and general impairment. Afterwards, an expected number of *N* = 140 patients, for whom routine clinical care has been proven to be insufficient and who still suffer from severe ADHD, will be randomised either to the IMTP or to TASH. Treatments will be administered over the course of 12 weeks (*step 2* of the study). Measurements will be taken at baseline (T0: screening of inclusion and exclusion criteria and assessment of ADHD symptoms; T1: baseline assessment comprising anamnestic information, pharmacological and non-pharmacological treatment history, additional information on comorbidity and parental self-ratings regarding child variables and own mental health), after the TAU phase (T2), after the treatment phase (T3) and 12 weeks after the end of the treatment (follow-up examination; T4). Patients failing to meet the severity criterion after TAU and not having entered the randomisation stage will also be invited for a follow-up appointment (T4) in order to monitor their progress in clinical routine treatment. Patients who drop out of the study will be encouraged to still participate in the next study visits for data collection. For an overview of the trial flow, please refer to Fig. 1. Clinical assessments of ADHD symptoms and comorbid psychopathology constituting outcome parameters will be completed by an experienced clinician who is blind to the patient’s assignment to treatment condition, but not to the time point (T0–T4). All randomisation decisions will be based on those semi-blinded ratings. All interviews will be recorded and subsequently a subsample will be rated by a blinded expert in order to validate the semi-blinded ratings by the clinician. Concerning safety of study participants, unblinding will not be necessary, since blinding is limited to outcome raters.

### Trial sites

Patients will be recruited at six sites across Germany. The leading centre coordinating the study is the Department of Child and Adolescent Psychiatry, Psychosomatics and Psychotherapy at the University Hospital of Würzburg. Other recruiting centres are the Departments of Child and Adolescent Psychiatry, Psychosomatics and Psychotherapy at the University Hospitals of Köln, Hamm, Tübingen, Mannheim (Central Institute of Mental Health) and Marburg. Each centre will enrol approximately 25 patients in order to achieve a total sample size of *N* = 160 adolescents. Changes in procedures or protocol amendments are communicated to all study centres via the Clinical Trials Unit at the University Medical Centre Freiburg and / or the coordinating centre.

### Participants

The study’s target group are adolescents with ADHD with insufficient improvement of symptoms through at least 1 year of treatment in routine clinical care. Patients will be recruited primarily through the centres’ outpatient clinics. Further recruitment strategies include dissemination of information regarding the study at local conferences, in schools and via contacting paediatricians and other care institutions. Baseline characteristics will be collected in an interview to assess basic information on medical history and sociodemographic information.

Adolescents are considered to be severely affected if they meet the criterion of high impairment ratings on the severity of symptoms scale of the *Clinical Global Impression scale* (CGI-S ≥ 4, [51]). Severity will be assessed during screening for general inclusion into the study and after the TAU phase. Children falling below the threshold for severe ADHD after TAU will not be randomised to one of the two behavioural interventions in step 2, but a follow-up assessment will be carried out. Patients will be included if they meet all of the eligibility criteria displayed in Table 1.

**Table 1:** Inclusion and exclusion criteria

Inclusion criteria	• Age 12;0 to 17;11 years • ADHD according to DSM-5 criteria, assessed with a structured clinical interview • Patient has been in ADHD routine care for a minimum of 6 months (does not have to be the year immediately preceding the study; routine care defined as treatment aimed at ADHD symptoms as prescribed or provided by a child and adolescent psychiatrist, psychotherapist or paediatrician) • No sufficient benefit from previous interventions developed for the treatment of ADHD, i.e. still substantial impairment (CGI-S ≥ 4) under current treatment • Patients and primary caregiver speak sufficient German for psychotherapy
Exclusion criteria	• IQ < 80, assessed with the Wechsler Nonverbal Scale of Ability [101, 102] • Comorbidity: pervasive developmental disorder, schizophrenia, bipolar disorder, severe depressive episode • Need for inpatient treatment • After TAU phase: intensive psychotherapy on a biweekly or more intensive basis • After TAU phase: psychopharmacological medication which is not constant or not licensed for the treatment of ADHD or comorbid conditions

### Data handling

All legal requirements regarding the protection of personal data will be met. Each participant will be assigned a study-specific identification code upon enrolment. All study data collected from patients will be stored under that code, ensuring complete pseudonymisation. This information will not be shared. The only exception is transmission of contact details to members of the University Hospital Köln who provide the TASH intervention with the participants’ consent. Access to the patient identification list is limited to the principal investigators (PIs) and the study coordinators at each site. The Clinical Trials Unit (CTU) Freiburg provides an electronic remote data entry system (RDE-LIGHT), where information is entered by specially trained personnel under the study code. Built-in security features encrypt all data before transmission to and from the CTU, thus preventing unauthorised access to confidential participant information. Users entering data into that system will be registered with the CTU with an individual ID and password to gain access to the system, thus preventing unauthorised access to patient data. Data processing at the CTU is limited to authorised personnel that is familiar with the data handling procedures according to the study protocol.

### Interventions

#### Individualised modular treatment programme (IMTP)

The IMTP as a focused, short-term, cognitive behavioural intervention consists of 10 weekly sessions of 60 min over a period of 12 weeks (for an overview, see Table 2). The contents of the opening and the closing sessions (sessions 1, 2, 9 and 10) are mandatory for all participants. Sessions 3–8 comprise the strongly individualised part of the treatment, where different modules can be chosen depending on the adolescents’ core problem areas for a focused and economical treatment of persistent difficulties. Individualisation is based on clinical data gathered at screening and baseline visits and goal setting of patient and therapist in sessions 1 and 2. Treatment is offered in individual sessions because the required degree of individualisation of treatment cannot be achieved in a short-term group psychotherapy setting. Sessions 1 and 2 focus on the establishment of the therapeutic relationship, psychoeducation, the development of an individual concept of the disorder and the selection of three focus modules for the individualised part of the therapy. Each module spans two treatment sessions. Sessions 9 and 10 aim at recapitulating and consolidating the contents of the previous sessions and planning the future treatment.

**Table 2:** Overview of the Individualised Modular Treatment Programme (IMTP) modules

	Title	Target	Participants
A	Organisation is key	Organisational skills and planning	A
B	Full concentration	Distractibility and procrastination	A
C	The Courage Module	Dysfunctional thinking	A
D	The Emotion Module	Emotion regulation	A
E	Less stress – greater satisfaction	Problem solving, stress management	A
F	The Medication Module	Medication management	A
G	Thrill seekers	Harmful substance (ab)use	A
H	Improving family communication	Dysfunctional communication	P + A
I	Parent training	Parental competence	P (+A)
J	Keeping an eye on own well-being	Parental mental health	P

*A* = adolescent, *P* = parent

#### Telephone-assisted self-help (TASH)

As active control treatment, 10 weekly sessions of TASH [50] are implemented (for an overview, see Table 3). TASH is based on established principles of behavioural parent training. It includes strategies aiming at enhancing positive parent-child-interactions and controlling hyperkinetic and oppositional behaviour (e.g. communicating demands effectively, setting positive and negative consequences consistently). TASH in the context of this study uses a bibliotherapeutic approach with brochures specifically developed for parents of adolescents with ADHD in former trials. A total of eight brochures will be sent to the parents and will be supplemented by 10 counselling sessions of about 30 min provided by trained psychologists and pedagogues via telephone in order to support the parent in the implementation of the recommendations presented in the brochures.

**Table 3:** Overview of telephone-assisted help programme (TASH) brochures for parents

	Title	Target
1	ADHD in adolescence	Psychoeducation regarding ADHD symptoms, associated problems, the courses of ADHD and treatment alternatives
2	Analysing and tackling problems	Analysing problems and coercive parent-child interactions; focus on strengths
3	‘With each other, not against each other’	Escaping coercive parent-child interactions; positive interactions; rules of communication
4	Re-evaluating rules	Reconsidering and defining rules; agreements for solving frequent conflicts
5	Joint negotiations	Holding constructive problem talks with adolescents
6	Planned consequences, step 1	Making clear demands and reinforcing positive behaviour
7	Planned consequences, step 2	Adequate negative consequences and behaviour contracts
8	Regenerating and looking to the future	Parental well-being and future prospects of the parents

#### Treatment integrity

Treatment integrity will be established through qualification standards for therapists (a university degree qualifying for training to become a licenced child and adolescent therapist, currently in training for psychotherapy with children and adolescents and clinical expertise in the treatment of ADHD), study-specific therapist training, the use of manualised treatment programmes, the use of protocol sheets for treatment documentation, video- and audio-taping of treatment sessions, adherence and integrity ratings, and structured video-based supervision for two out of 10 treatment sessions and feedback.

Concomitant treatment is permitted if it is not considered intensive behavioural therapy (≥ bi-weekly). Psychopharmacological treatment for ADHD is allowed. Changes in medication during the treatment phase are discouraged except when clinically necessary. If participants clearly need additional treatment besides the study interventions, add-on treatments may be discussed and participation in the study may be terminated by the study team.

### Primary and secondary outcome measures

#### Primary outcome

The primary endpoint is the change in ADHD symptoms from T2 to T3 measured by changes in the total score of the Diagnose-Checkliste für Aufmerksamkeitsdefizit−/Hyperaktivitätsstörungen (DCL-ADHS), a structured DSM-5-based clinical interview. It comprises 18 items assessing ADHD symptoms, and five items assessing functioning and psychological strain and shows good internal consistency (Cronbach’s α = 0.89–0.95) and high validity [52–54]). All items are rated on a 4-point scale (0–3; symptom present if ≥2). For ESCAadol, the interview is conducted with parents and adolescents in a joint session.

#### Secondary outcomes

The most important secondary endpoint is the change on the Clinical Global Impression Scale (CGI, [51]) from T2 to T3 and from T2 to T4. It measures the severity of the disease (CGI-S) and the general improvement through treatment (CGI-I). The assessment takes place after a short clinical interview. CGI-S and CGI-I are evaluated on a 7-point scale, with higher scores indicating a greater severity or impairment, respectively. Despite conflicting findings with regard to reliability and validity, the CGI is widely used in clinical trials as an outcome parameter. The CGI-I shows good interrater reliability (0.65–0.92) [55] and an intra-class correlation coefficient of 0.91 [56].

Further secondary endpoints are changes in patient-, parent- and teacher-rated ADHD and symptoms of oppositional defiant disorder (ODD) and conduct disorder (CD), ADHD-related functional impairment and quality-of-life, internalising and externalising symptoms and parenting from T2 to T3, and T2 to T4:

#### ADHD and ODD/CD symptoms

Parents, teachers and patients rate the severity of all of the core symptoms of ADHD and a core set of symptoms of CD on a 4-point scale on the questionnaires *Fremdbeurteilungsbogen für Aufmerksamkeitsdefizit-/Hyperaktivitätsstörungen* (FBB-ADHS; parent report Cronbach’s α = 0.91–0.94; teacher report Cronbach’s α = 0.87–0.93), *Selbstbeurteilungsbogen für Aufmerksamkeitsdefizit-/Hyperaktivitätsstörungen* (SBB-ADHS; self-report; Cronbach’s α = 0.79–0.89), *Fremdbeurteilungsbogen für Störungen des Sozialverhaltens* (FBB-SSV; parent report Cronbach’s α = 0.67–0.91; teacher report Cronbach’s α = 0.69–0.92) and *Selbstbeurteilungsbogen für Störungen des Sozialverhaltens* (SBB-SSV; self-report [52]; Cronbach’s α = 0.65–0.89). The Diagnose-Checkliste für Störungen des Sozialverhaltens (DCL-SSV) will be completed by a semi-blinded clinician rater based on an interview with parents and adolescents [52]. This interview also shows good internal consistency (Cronbach’s α = 0.68–0.88).

#### Functional impairment and quality of life

The *Weiss Functional Impairment Rating Scale* (WFIRS-P, [57, 58]) assesses impairment specifically associated with ADHD. Parents rate 40 items on the dimensions family, learning and school, life skills, child’s self-concept and social activities on a 4-point scale. This interview also possesses good internal consistency (Cronbach’s α > 0.7), test-retest reliability (*r* > 0.7) and validity [59, 60]. The *Health-Related Quality of Life Questionnaire for Children and Young People* (KIDSCREEN-10, [61, 62]) comprises 10 items and measures the parent- and child-rated subjective health and well-being of children and adolescents on a 5-point scale. The authors report good internal consistency (Cronbach’s α = 0.82) and test-retest reliability (*r* = 0.73).

#### Internalising and externalising symptoms

The *Child Behaviour Checklist* (CBCL/6-18R) is a parent questionnaire assessing the behavioural and emotional problems of children and adolescents with 113 items on a 3-point scale. The instrument shows good reliability for most scales (rtt > .80) and good validity [63–65].

#### Parenting Rating Scales

Perceived parenting sense of competency concerning difficult parenting situations is assessed with the questionnaire *Verhalten in Risikosituationen* (VER [66]). The first 14 items describe typical situations (e.g. going shopping). Thirteen other items describe child behaviour (e.g. ‘refuses to eat’). On a 4-point scale, parents can state how well they can manage the situations. Positive parenting will be assessed with the *Fragebogen zum Erziehungsverhalten* (FZEV), which has been devised using items from the Positive Parenting subscale of the *Parent Practices Scale* (PPS [67]) among others. It consists of 13 items which measure positive, reinforcing and encouraging parental behaviour such as praise, playing together, attention and physical affection on a 4-point scale. Both instruments show good internal consistency [67, 68] and have been used in multiple intervention studies; e.g. [69]. Negative parenting is measured by a short version of the negative parenting subscale of the German questionnaire *Fragebogen zum positiven und negativen Erziehungsverhalten* (FPNE [70]. The questionnaire consists of 13 items with a 4-point scale.

### Therapy preconditions and process variables

To gain insight into motivational aspects for treatment as well as treatment quality, measures of treatment expectation, motivation and satisfaction ratings of participating parents and the adolescents are included. Therapists will rate their treatment expectation as well as provide data regarding treatment integrity and adherence. Furthermore, we will examine changes in the concentration of genetic transcripts such as micro-RNAs and mRNAs in peripheral tissue as potential biological correlates of response to therapy pre- and post-treatment.

### Predictors and moderators of therapeutic outcome

#### Predictors – Psychometric data

As possible predictors of the primary treatment outcome, we will collect data with regard to sociodemographic information (e.g. age and sex, IQ, familial psychosocial risk factors) and treatment expectation of the parent, adolescent and therapist.

Furthermore predictors comprise child temperament (*Junior Temperament and Character Inventory,* JTCI; parent- and self-report; test-retest reliability *r* = 0.65–0.87; Cronbach’s α = 0.79–0.85; validity [71–73]), emotional dysregulation (adolescent self-report on the German questionnaires *Fragebogen zur Erhebung der Emotionsregulation bei Kindern und Jugendlichen,* FEEL-KJ; test-retest reliability *r* = 0.62–0.81; Cronbach’s α = 0.69–0.91; validity [74, 75]), and social responsiveness of the child (*Social Responsiveness Scale,* SRS; test-retest reliability *r* = 0.72–0.91; Cronbach’s α = 0.91–0.97; validity [76–79]), problem behaviour on various domains according to the *Youth Self Report Scale* (YSR; good test-retest reliability internal consistency and validity [80–82]) and irritability (*Affective Reactivity Index,* ARI; test-retest correlation *r* = 0.88 for parent-report and 0.29 for self-report; Cronbach’s α = 0.89 for parent-report and 0.90 for self-report [83]; validity [84]).

Furthermore we assess parental variables such as ADHD (*ADHS-Selbstbeurteilungsskala* ADHS-SB: test-retest reliability *r* = 0.78–0.89; Cronbach’s α = 0.72–0.9, validity [85]*; Wender Utah Rating Scale – deutsche Kurzform,* WURS-K: test-retest reliability *r* = 0.9; Cronbach’s α = 0.91, validity [85, 86]), depression, anxiety and stress (*Depression Anxiety Stress Scales,* DASS; Cronbach’s α = 0.89–0.96, test-retest realability *r* = 0.71–0.81, validity [87, 88]) as well as parental coping with anger (*Elternfragebogen zum Umgang mit Ärger*, FB-Ä [89]) with self-report questionnaires. As a further predictor, clinical rating scores on psychiatric comorbidity in the child obtained with the structured screening interview *Diagnose-Checkliste zum Screening psychischer Störungen* (DCL-SCREEN*,* [52]) will be used. In this study, the DCL-SCREEN will be used to screen for symptoms of autism spectrum disorders, depressive disorders, anxiety disorders, obsessive-compulsive and related disorders, tic disorder, somatoform and related disorders, motor and language disorders, specific learning disorders and elimination disorders. In case of clinical symptoms emerging in the *DCL-SCREEN*, we will use the diagnostic checklist for the particular disorder from the *Diagnostik-System für psychische Störungen nach ICD-10 und DSM-5 für Kinder- und Jugendliche* (DISYPS-III*,* [52]) for an extensive assessment based on DSM-5 diagnostic criteria.

#### Predictors – Biological variables

The selected predictors will be obtained through assessments with electroencephalography (EEG; spontaneous and event-related EEG-recordings), magnetic resonance imaging (structural MRI, diffusion tensor imaging and functional MRI) as well as transcranial sonography (TCS). Furthermore, blood and saliva samples will be collected from all participants before (T1) and after treatment (T3). EEG and MRI measurements are limited to the subgroup receiving the IMTP and will be performed following randomisation. TCS measurements will be performed at any time during the study with all patients who provide informed consent.

TCS is a non-invasive method for the visualisation of deep brain structures through the intact skull. Ultrasound waves are reflected depending on tissue composition, resulting in different echogenicity of nuclei and ventricular system. Of particular interest is the mesencephalic scanning plane including brainstem, substantia nigra and raphé nuclei. In children, ADHD-associated hyperechogenicity of the substantia nigra has consistently been reported and it has been proposed as a potential biological marker for ADHD [90, 91]. TCS can aid differential diagnosis (e.g. in movement disorders, [92]) and has shown promise in the prediction of treatment response in psychiatric disorders in adult patients [93, 94]. As of yet, no study has explored the possibility of using TCS to predict the effectiveness of non-pharmacological interventions. In the context of our study, the TCS-based predictor of treatment outcome will be the size of the echogenic area of the substantia nigra, which has been shown to be associated with the disorder [90, 91].

Genetic material will be extracted from blood and saliva samples. We will analyse genetic variants in candidate gene systems for their predictive value regarding an individual’s response to different therapeutic interventions.

The EEG-based predictors consist of the frequency profile at rest (spontaneous theta-band and alpha-band activity) and the strength of preparatory cognitive activity (contingent negative variation amplitude), which explain nearly 30% of the variability in behavioural improvement following neurofeedback treatment [95]. To test the mechanisms of improvement underlying the IMTP, EEG recordings are repeated following this treatment in step 2. The changes in brain electric activity, i.e. the hypothesised reduction of resting theta-activity and increased contingent negative variation activity, will be treated as additional secondary outcome measures in this subgroup.

The MRI-based predictors consist of the integrity (fractional anisotropy) of the fronto-striatal connection and of volumetric grey matter density of the implicated dorsolateral-prefrontal und striatal regions.

As neuropsychological predictors we include four computer-based experimental paradigms: The *continuous performance task* (CPT-OX, [96]) measures selective attention and impulsive behaviour by requiring participants to withhold a prepared response through inhibitory response control. The *monetary incentive delay task* (MID, [97, 98]) is a paradigm to study reward anticipation and reward feedback in an event-related task with three task conditions (no win; small win; big win). The S*top Signal Task* (SST, [98, 99]) is used to study inhibitory control. The task composes go-trials and stop-trials. A tracking algorithm changes the time interval between go-signal and stop-signal onsets according to each subject’s performance on previous trials.

#### Moderators

For the investigation of moderators of treatment outcome, we will examine the influence of age, sex, socioeconomic status, ADHD symptom severity, comorbid symptoms, intelligence, parental mental health (parental depression, anxiety and stress assessed by self-ratings on the DASS; parental ADHD assessed with the ADHD-SB).

**Fig. 2:**
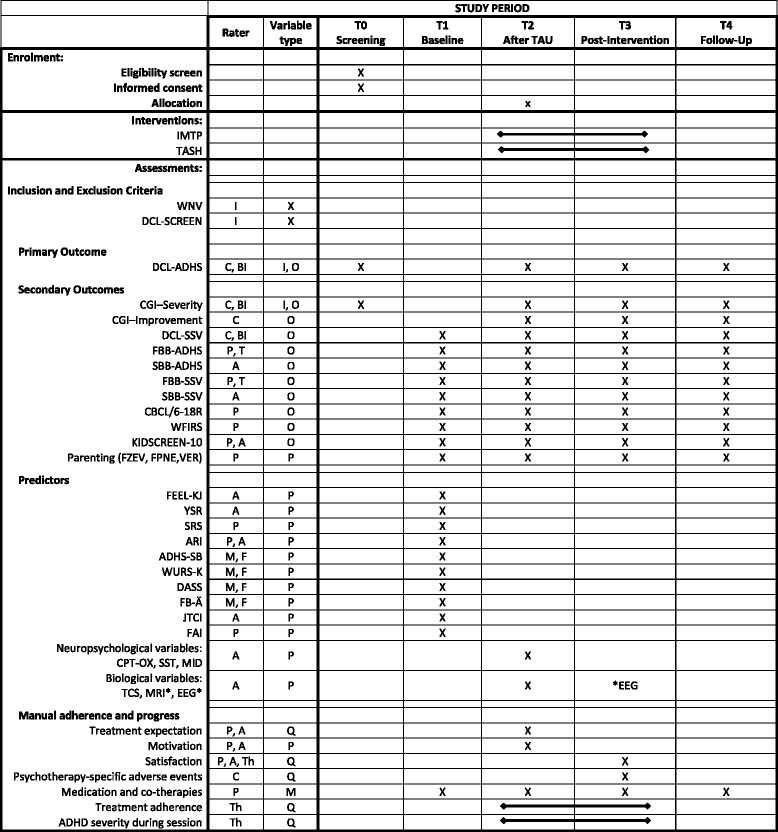
Overview of outcome measures, predictors and eligibility criteria. Rater: *A* = adolescent, *B* = blind rater, *C* = clinician, *F* = father, *M* = mother, *P* = parents, *T* = teacher, *Th* = therapist; Variable type: *I* = inclusion criterion, *O* = outcome variable, *P* = predictor, *Q* = variable for quality control; Interventions, *IMTP* = Individualised Modular Treatment Programme, *TASH* = telephone-assisted self-help; measures: *ADHS-SB* = ADHS-Selbstbeurteilungsskala, *ARI* = Affective Reactivity Index, *CBCL* = Child Behaviour Checklist, *CGI* = Clinical Global Impression, *DASS* = Depression Anxiety Stress Scales, *DCL-ADHS* = Diagnose-Checkliste für Aufmerksamkeitsdefizit−/Hyperaktivitätsstörungen, *DCL-SCREEN* = Diagnose-Checkliste zum Screening psychischer Störungen, *DCL-SSV* = Diagnose-Checkliste für Störungen des Sozialverhaltens, *EEG* = electroencephalogram, *FAI* = Family Adversity Index, *FB-Ä* = Elternfragebogen zum Umgang mit Ärger, *FBB-ADHS* = Fremdbeurteilungsbogen für Aufmerksamkeitsdefizit-/Hyperaktivitätstörungen, *FBB-SSV* = Fremdbeurteilungsbogen für Störungen des Sozialverhaltens, *FEEL-KJ* = Fragebogen zur Erhebung der Emotionsregulation bei Kindern und Jugendlichen, *FPNE* = Fragebogen zum positiven und negativen Erziehungsverhalten, *FZEV* = Fragen zum Erziehungsverhalten, *JTCI* = Junior Temperament and Character Inventory, *KIDSCREEN-10* = The Health-Related Quality of Life Questionnaire for Children and Young People, *MID* = Monetary-Incentive-Delay-Task, *MRI* = magnetic resonance imaging*, SBB-ADHS* = Selbstbeurteilungsbogen für Aufmerksamkeitsdefizit-/Hyperaktivitätstörungen, *SBB-SSV* = Selbstbeurteilungsbogen für Störungen des Sozialverhaltens, *SRS* = Social Responsiveness Scale, *SST* = Stop Signal Task, *TCS* = transcranial sonography, *VER* = Verhalten in Risikosituationen, *WFIRS-P* = Weiss Functional Impairment Rating Scale, *WURS-k* = Wender Utah Rating Scale – short version, *YSR* = Youth Self Report Scale. *only for patients randomised to one of the two treatment conditions

For an overview of all measures used at individual time points throughout the study, please refer to Fig. 2.

### Rater training

Semi-blinded clinicians and blinded raters will be trained in the assessment of ADHD, CD and comorbid conditions with the DCL-ADHS and DCL-SSV. After viewing and scoring a video recording of a diagnostic interview using the DCLs, their ratings will be compared to a ‘gold standard’ established by the authors of the DISYPS-III. The first three interviews with participants will be supervised by a certified rater. A reliability analysis will be conducted at the end of the study.

### Randomisation procedure

Central randomisation with a 1:1 treatment ratio will be performed by the CTU at the University Medical Centre Freiburg via fax, using block randomisation with variable block length to guarantee concealment of randomisation. Randomisation will be stratified by centre. The randomisation request form contains the following information: study-specific patient identification number, year of birth and the confirmation of ADHD > cut-off. The central randomisation office will review the patient’s details on the randomisation fax. If data on the fax are complete and appropriate, the randomisation will be performed by the central randomisation office. The patient will be entered in the randomisation list at the randomisation office, and the randomised treatment arm will be sent to the investigator by fax. The returned randomisation fax will contain the information entered by the investigator complemented by the information about the randomised treatment (IMTP or TASH).If the details on the randomisation fax appear to be incomplete or implausible, the central randomisation office will send a query fax to the investigator for clarification.

### Monitoring and safety

The monitoring is performed by the clinical research associates (CRAs) of the CTU. Adapted monitoring will be done according to Good Clinical Practice (ICH-GCP) and standard operating procedures (SOP) to verify that patients’ rights and well-being are protected, reported trial data are accurate, complete and verifiable from source documents and that the trial is conducted in compliance with the currently approved protocol/amendment, with ICH-GCP and with the applicable regulatory requirements to ensure safety and integrity of clinical trial data. Prior to the trial, a pre-trial visit by phone, a site initiation visit at each site and an investigators’ meeting are conducted in order to train and introduce the investigators and their staff to the trial protocol, essential documents and related trial specific procedures, ICH-GCP and national/local regulatory requirements. Additional training via telephone may be employed if necessary.

During the trial, the monitor will visit the site regularly depending on the recruitment rate and quality of data. During these on-site visits, the monitor verifies that the trial is conducted according to the trial protocol, trial specific procedures, ICH-GCP and national/local regulatory requirements. The presence of signed informed consents, the eligibility of patients, primary endpoint, treatment compliance and documentation will be verified by the monitor. The monitor is also performing source data verification to ensure that clinical trial data are recorded and documented in the source data and case report forms (CRFs) are complete and accurate. Extent of source data verification and monitor visit frequency will be adapted for individual sites in case of lack of data quality or a high number of protocol violations.

An independent Data Monitoring Committee (DMC) is formed by Prof. Dr. med. H. J. Freyberger, Prof. Dr. med. A. Rothenberger and Prof. Dr. med. J. Schmitt. The DMC will advise the trial sponsor on patient safety and measures to ensure the credibility and integrity of the ongoing trial.

### Stopping rules

Stopping rules for an individual patient include withdrawal of informed consent of parents / guardians or the patient, need for inpatient treatment or other reasons affecting the patient’s well-being in the case of continued trial participation or need for a different kind of treatment for health reasons according to the judgement of the attending physician. The Ethics Committee will be informed immediately in case of severe adverse events during the conduct of the trial. Global stopping rules for the trial or closing of a centre include emerging of data leading to a revision of the risk-benefit ratio, on-going failure of recruitment or repeated violations of the study protocol or standard GCP rules. For a decision on the termination of the trial or of closing a centre, agreement between PI, site investigators, DMC members, responsible Ethical Committee and the CTU Freiburg is intended.

### Proposed sample size and power calculations

The calculation of the sample size (software: STPLAN Version 4.3) is based on the primary endpoint ‘change in total score of DCL-ADHS from T2 (after TAU-stabilisation) to T3 (after experimental or control treatment; 12 weeks)’ using the two-sided t test with a power of 80% at a significance level of 5%. Studies on psychosocial interventions on the treatment of ADHD report effect sizes of about *d* = 0.8 [100], where *d* denotes the difference in the change score between the two randomised groups in standard deviation units. However, we have chosen a smaller effect size to calculate the sample size because recent meta-analyses referring to less biased outcome criteria such as assessments by clinicians report smaller effects of non-pharmacological interventions for the treatment of ADHD [22]. Furthermore, these effect sizes are based on trials with mostly waiting control group, thus, we expect our effects to be smaller. Additionally, in our highly affected clinical sample treatment effects might be less pronounced. A smaller effect size also accounts for the fact that the planned intention-to-treat approach might dilute the true expected effect – patients who discontinue therapy will be included in the primary analysis. We assume low to zero pre-post effects within our control group. Therefore, an effect size of *d* = 0.5 between groups seems realistic, and it is considered to be still clinically relevant. To detect a difference assuming the true effect size is *d* = 0.5, 64 patients with non-missing data per group are required (critical *t* = 1.98, *df* = 126). In order to account for the possibility that a few patients (8%) will have incomplete data at T3, in total 140 patients will have to be randomised. Studies on psychosocial interventions in ADHD children and their parents report attrition rates ranging from 5 to 20%. The study by Antshel et al. on CBT for adolescents with ADHD-symptoms unresolved by medication fits best with our intended sample. In this trial CBT adherence was good (all patients attended 13–16 sessions with half of the patients never missing an appointment and half missing between one and three appointments). Therefore, a rate of 8% with incomplete data at T3 seems conservative [23].

The sample size will provide sufficient power to handle the described imponderabilia of dropouts and incomplete data. Based on clinical experience we assume that about 10% of the patients will be improved after TAU-stabilisation, and, therefore, estimate that *N* = 160 need to be recruited at baseline to attain 140 randomisations. Based on our prior research on clinical trials in ADHD [33] we expect a 30% to 40% rate of screening failures. Thus, about *N* = 250 patients will need to be screened for study participation.

### Statistical analyses

All statistical programming for analysis will be performed with the Statistical Analysis System (SAS®).The primary analysis is based on an intention-to-treat approach. This means that the patients will be analysed in the treatment arms to which they were randomised, irrespective of whether they refused or discontinued the treatment or whether other protocol violations are revealed. The per-protocol (PP) population is a subset of the Full Analysis Set (FAS) and is defined as the group of patients that had no major protocol violations, received a predefined minimum dose of the treatment and underwent the examinations required for the assessment of the endpoints at relevant, predefined times. The analysis of the PP population will be performed for the purpose of a sensitivity analysis.

Safety analyses will be performed in the safety population. Patients in the safety population are analysed as belonging to the treatment arm defined by treatment received. Patients are included in the respective treatment arm, if treatment was started / if they received at least one dose of trial treatment.

#### Analysis of primary endpoint

The primary statistical analysis will be by intention-to-treat, so that all randomised patients will be analysed according to their allocated arm. Changes in the DCL-ADHS score from T2 to T3 and T2 to T4, respectively, will be evaluated in a linear regression model including treatment, centre, visit and the respective T2-baseline DCL-ADHS. Further covariates predictive of missingness will be included based on a pre-specified selection strategy to correct for potential bias arising from missing data. The primary treatment comparison of the change score at T3 will be based on least-squares means with a two-sided 95% confidence interval. Other possibly relevant covariates may be considered as well. Subgroup analyses will be conducted in an exploratory manner by inclusion of interaction terms in the linear regression model. They will focus on the analysis of patients’ and parents’ comorbidities. In addition, gender effects will be investigated as prognostic and predictive factors.

#### Analysis of secondary endpoints

Secondary endpoints will be analysed descriptively in a similar fashion as the primary outcome, using regression models as appropriate for the respective type of data. Treatment effects will be calculated with two-sided 95% confidence intervals. Details are specified in a statistical analysis plan which was prepared before the inclusion of the first patient. For all endpoint scores the change between T2-baseline and T3, and T2 and T4 will be evaluated. The analysis of the change between T2-baseline and T3 will be done similar to the analysis of the primary endpoint for continuous measurements. The difference in CGI between T2 and T3 will be analysed using the Mann-Whitney-*U*-test. The difference in CGI between T2 and T4 will be analysed using a Kruskall-Wallis test. Possible moderators and mediators of the DCL-ADHS score will be analysed using linear regression.

## Discussion

Adolescence is a crucial period for determining patients’ life trajectories in terms of mastering educational and psychosocial developmental tasks. However, in adolescents with ADHD motivation to seek treatment, treatment adherence and compliance are often lacking [15]. Furthermore, comorbidities gain importance; however, this is often not adequately addressed by standard psychological and pharmacological interventions for ADHD. Psychopharmacological treatment is of limited effect in this age group [12, 14]. Thus, there is a strong need for effective psychosocial interventions that generate high motivation and compliance and that also take into account non-core ADHD symptoms, such as emotion regulation and self-image, as well as comorbidity. Considering the limited empirical evidence for non-pharmacological treatment options of ADHD in adolescence, ESCAadol is conceptualised as a RCT for the evaluation of an innovative short-term CBT programme tailored to the needs of ADHD patients aged 12 to 17 years. One limitation of the trial is the absence of a waiting control group under routine clinical care. However, considering the limited number of potential participants in that age group, limiting the study design to two groups seemed necessary for obtaining a sufficient sample size. Therefore, we decided on an active control treatment to provide at least minimal support for adolescents in that crucial period for ethical reasons. Furthermore, demonstrating superiority of a short-term behavioural therapy programme over an active control condition presents a more rigorous test of the treatment efficacy than comparing it to no intervention. This RCT will be conducted in accordance with the highest standards of psychotherapy research, employing blinded outcome ratings and relying on power analyses to ensure adequate statistical power to detect effects. All raters and therapists receive special training prior to being involved with the study. The main treatment comprises a manualised therapy. Adherence to the manual will be checked. We compare the efficacy of this adolescent-centred intervention with a parent-centred telephone-assisted self-help programme as an active control treatment. Taking into account the broad spectrum of clinical presentations, comorbidities and environmental factors, the study furthermore aims at achieving a sample size that allows for the identification of predictors of treatment response across different modalities (psychometric variables, neuropsychological tests, brain functioning during rest and cognitive activity, structural brain imaging and biomarkers in blood and saliva). Results aim to fill a knowledge gap in the treatment of adolescents with ADHD who do not benefit from routine clinical care and at evaluating a cost-effective and feasible individualised treatment that can easily be implemented in routine clinical practice within the context of a personalised medicine approach.

### Trial status

Recruitment for this trial is ongoing.

## Additional file

Additional file 1: SPIRIT 2013 Checklist: recommended items to address in a clinical trial protocol and related documents*. (DOC 126 kb)

Additional file 2: World Health Organization Trial Registration Data Set. (DOC 17.4 kb)

## Abbreviations

ADHD: Attention-deficit/hyperactivity disorder
ADHS-SB: German self-rating scale ‘ADHS-Selbstbeurteilungsskala’ to assess parental ADHD
ARI: Affective Reactivity Index
BAS: Behavioural Activation Systems
BAT: Behavioural Avoidance Test
BIS: Behavioural Inhibition Systems
BT: Behavioural therapy
CBCL/6–18: Child Behaviour Checklist for ages 6–18 years
CBT: Cognitive behavioural therapy
CD: Conduct disorder
CGI: Clinical Global Impression
CNV: Contingent negative variation
CPT-OX: Variant of the continuous performance task
CRA: Clinical research associate
DASS: Depression Anxiety Stress Scales
DCL-ADHS: German clinical checklist ‘Diagnose-Checkliste Aufmerksamkeitsdefizit-/Hyperaktivitätsstörung’ to assess ADHD symptoms and impairment
DCL-SSV: German Clinical Checklist ‘Diagnose-Checkliste für Störungen des Sozialverhaltens’ to assess symptoms and impairment of disruptive behaviour disorders and related problems
DMC: Data Monitoring Committee
DRKS: German Clinical Trials Register
DSM-5: *Diagnostic and Statistical Manual of Mental Disorders, Fifth Edition* (DSM-5), Classification and diagnostic tool for mental disorders issued by the American Psychiatric Association
EEG: Electroencephalography or electroencephalogram
ESCAadol: Acronym for the study ‘Evidence-based stepped care of ADHD: individualised short-term therapy for adolescents impaired by ADHD despite previous routine care treatment’
FAI: Family Adversity Index
FAS: Full Analysis Set
FB-Ä: German questionnaire ‘Elternfragebogen zum Umgang mit Ärger’ to assess parental aggression
FBB-ADHS: German Observer Rating Scale ‘Fremdbeurteilungsbogen für Aufmerksamkeitsdefizit /Hyperaktivitätsstörung’ to assess ADHD symptoms and impairment
FBB-SSV: German Observer Rating Scale ‘Fremdbeurteilungsbogen für Störungen des Sozialverhaltens’ to assess symptoms and impairment of disruptive behaviour disorders and related problems
FEEL-KJ: Questionnaire for the assessment of emotional dysregulation in children and adolescents
FPNE: German questionnaire ‘Fragebogen zum positiven und negativen Erziehungsverhalten ’ to measure negative parenting practices
FZEV: German questionnaire ‘Fragen zum Erziehungsverhalten’ to measure positive, reinforcing and promotive parenting practices
ICH-GCP: Good Clinical Practice
IMTP: Individualised Modular Treatment Programme, short-term behaviour therapy programme comprising 10 sessions specifically developed for adolescents with ADHD
IQ: Intelligence quotient
JTCI: German questionnaire ‘Junior Temperament und Charakter Inventar’ to assess child personality
KIDSCREEN-10: The Health-Related Quality of Life Questionnaire for Children and Young People
MID: Monetary incentive delay task
MMRM: Mixed-effects model for repeated measures
MRI: Magnetic resonance imaging
ODD: Oppositional defiant disorder
PP: Per protocol population
RCT: Randomised controlled trial
SBB-ADHS: Selbstbeurteilungsbogen für Aufmerksamkeitsdefizit-/Hyperaktivitätstörungen
SBB-SSV: Selbstbeurteilungsbogen für Störungen des Sozialverhaltens
SOP: Standard operating procedures
SRS: Social Responsiveness Scale
SST: Stop Signal Task
STAND: Supporting Teens’ Academic Needs Daily
Step 1: Observational period of 4 weeks
Step 2: Main treatment period with a duration of 12 weeks
T0: Screening at the beginning of the study to assess inclusion and exclusion criteria
T0/T1: To indicate overlap in diagnostic procedure of T0 and T1 assessment
T1: Assessment at baseline before Step 1
T2: Assessment at the end of Step 1 and before beginning of Step 2 intervention
T3: Assessment at the end of Step 2 intervention
T4: Follow-up assessment 12 weeks after the end of Step 2 intervention
TASH: Telephone-assisted self-help for parents
TAU: Treatment as usual, observation phase between T1 and T2
TCS: Transcranial sonography
VER: German questionnaire ‘Verhalten in Risikosituationen’ to assess the perceived ability to solve difficult parenting situations
WFIRS-P: Weiss Functional Impairment Rating Scale – parent report
WNV: Wechsler Nonverbal Scale of Ability, tool for assessment of the inclusion criterion intelligence level
WURS-K: Wender Utah Rating Scale, short version
YSR: Youth Self Report Scale
