# Supplementary material for: Individualised short-term therapy for adolescents impaired by attention-deficit/hyperactivity disorder despite previous routine care treatment (ESCAadol)—Study protocol of a randomised controlled trial within the consortium ESCAlife
Source: Trials. 2018 Apr 27;19:254. doi: 10.1186/s13063-018-2635-2 (PMC5921777; doi:10.1186/s13063-018-2635-2)
Supplement: Supplementary file 2 — World Health Organization Trial Registration Data Set. (DOC 17.4 kb) [file 13063_2018_2635_MOESM2_ESM.docx]

Table 1

*World Health Organization Trial Registration Data Set*

| **Data category** | **Information** |
| --- | --- |
| Primary Registry and Trial Identifying Number | German Clinical Trials Register (DRKS), DRKS00008974 |
| Date of Registration in Primary Registry | 28.12.2015 |
| Source(s) of Monetary or Material Support | Government funding body: Federal Ministry of Education and Research (BMBF), Friedrichstraße 130 B, 10117 Berlin, Germany |
| Primary Sponsor | Universitätsklinikum Würzburg, Josef-Schneider-Str. 2, 97080 Würzburg, Germany |
| Contact for Public Queries | MR, TJ, JG |
| Contact for Scientific Queries | MR, TJ, JG |
| Public Title | Individualized short-term therapy for adolescents impaired by ADHD despite previous routine care treatment (ESCAadol) |
| Scientific Title | Individualized short-term therapy for adolescents impaired by ADHD despite previous routine care treatment (ESCAadol) - a randomised controlled trial within the consortium ‘ESCAlife’ |
| Countries of Recruitment | Germany |
| Health Condition(s) or Problem(s) Studied | Attention-Deficit/Hyperactivity Disorder (ADHD) |
| Intervention(s) | Short term cognitive behavioural therapy, telephone-assisted self help |
| Key Inclusion and Exclusion Criteria | - Inclusion criteria: age 12-17 years; ADHD according to DSM-5 criteria; patient in ADHD routine care for minimum of 6 months ; no sufficient benefit from previous interventions (insufficient benefit defined as significant impairment by ADHD and/or co-morbid symptoms: CGI-S > 4); patients and primary caregiver speak sufficient German - Exclusion criteria: IQ < 80; comorbid pervasive developmental disorder, schizophrenia, bipolar disorder or severe depressive episode; need for inpatient treatment; co-treatment including intensive psychotherapies on biweekly or more intense basis or medication which is not constant or not licensed for the treatment of ADHD and co-morbid conditions |
| Study Type | Interventional; Allocation: Randomised; Intervention model: parallel assignment, Masking: blind |
| Date of First Enrollment | January 2016 |
| Sample Size | 160 |
| Recruitment Status | Recruiting |
| Primary Outcome(s) | Change in blinded clinician-rated ADHD-Checklist scores |
| Key Secondary Outcomes | Change on Clinical Global Impression Scale, symptoms of ADHD (patient-, parent- and teacher-rated) and oppositional-defiant and conduct disorder (patient-, parent-, teacher- and clinician-rated), ADHD-related functional impairment, internalizing and externalizing symptoms, quality-of-life and parenting |
| Ethics Review | Approved by the ethics committee of the medical faculty at the university of Würzburg (141/15) |

ADHD = Attention-Deficit/Hyperactivity Disorder; BMBF = Bundesministerium für Bildung und Forschung [Federal Ministry of Education and Research]; DRKS = Deutsches Register Klinischer Studien [German Clinical Trials Register], ESCAadol = “Evidence-based stepped care of ADHD: individualized short-term therapy for adolescents impaired by ADHD despite previous routine care treatment
